# From physical to metaversal events: An exploratory study

**DOI:** 10.1007/s43039-023-00068-1

**Published:** 2023-02-24

**Authors:** Niccolò Piccioni

**Affiliations:** Department of Communication and Social Research (CORIS), Sapienza University of Rome, Via Salaria, 113, Rome, Italy

## Abstract

The present study attempts to explore the meaning of the words “digital,” “virtual,” “hybrid,” “phygital,” “metaversal,” and “physical” applied to planned events. The primary aims is to understand whether there are significant differences among them and how managers can use them to achieve their objectives. The research identifies the projective techniques as the most appropriate method to underpin the phenomenon, and it adopts an exploratory-qualitative approach. Projective techniques appear innovative since they are frequently used for studying people’s instead of managers’ perspectives. The results allow us to classify the six dimensions of digitally transformed planned events as different, and a continuum is generated through the collected data. Finally, the article provides managerial suggestions, such as the pros and cons of each event category. For example, strategists organizing metaversal events should consider analyzing targets and platforms.

## Introduction

Technological progress is driving society transformations, and in particular in how companies think about, produce, distribute, and dispose of their product offerings (Heavin & Power, 2018; Verhoef et al., 2021). Digital transformation is affecting both goods and services (Hinings et al., 2018; Frank et al., 2019), and the latter are being revolutionized, including the effect of technology in incentivizing value capture and creation (Zaki, 2019).

One of the industries most affected by digital transformation is the planned events industry. Planned events (PEs) are unique spatial-temporal phenomena that are based on the interaction between people, setting and management, in order to generate an extraordinary experience (Getz, 2008). PEs are essential to companies’ marketing strategies, enabling engaging touchpoints with consumers and customers (Kotler & Keller, 2016).

PEs managers, responsible for delivering events, combine many digital and analogic tools in order to entertain, create the event experience and achieve marketing goals (Kotler & Keller, 2016). This is not new. In fact, since the time of the ancient Romans, technological innovations (such as fountains, fire games and gunpowder) gave ceremonialists the ability to convey unique emotions to everyone, regardless of their social status.

Many centuries later, since the spread of the Internet, PEs have faced a drastic revolution: the world has witnessed a profound transformation, and PEs moved from physical experiences to contaminated and hybridized environments (Getz & Page, 2020; Seraphin, 2021). In fact, since the beginning of the new millennium, event managers have started to deliver innovative experiences. For example, Momentum Agency (an Australian events agency) created for American Express in 2010 (see https://www.momentumww.com/project/we-pioneer-streaming-concerts/), the first “digital,” “streaming,” and “virtual” concert series, engaging more than 100 million people online (YouTube, Vimeo, and real-time chat on the website) and involving American Express customers who were invited to attend the physical concert. PianoB (an Italian event agency) and Amigdala (a Swiss event agency) cooperated to create a “contaminated” experience for Sorgenia in 2019 (see https://www.amigdala.ch/project/your-love-energy). There, people were invited to sit in front of a three-camera system that, after reading emotions and facial expressions, could change the setting (lights, sounds, etc.) of the physical venue. In this vein, over the years, many words have been used to define digitally transformed events, such as “digital”, “virtual”, “hybrid”, and “phygital”. Nowadays, also the term “metaverse” entered in the PEs vocabulary.

However, despite the terms’ heterogeneity, no integrated comparison has ever been made to understand the differences between virtual, hybrid, digital, phygital and metaversal PEs; indeed, these terms seem, sometimes, equivalent. In fact, some of the above words are used interchangeably by both scholars and managers, and there is no classification of them. Thus, the study aims to fill this gap by replying to the following research question:

*What are the characteristics of, and the differences and similarities between, “physical”, “digital”, “virtual”, “hybrid”, “phygital”, and “metaverse” events?*

Thus, the research intends to simultaneously (a) singularly investigate and (b) compare “digital,” “virtual,” “hybrid,” “phygital,” “metaversal,” and “physical” PEs to understand if there are significant differences and similarities between them. The study is shown to be relevant considering two aspects: on the one hand, the absence of a similar classification; on the other hand, the desire to offer greater clarity on the phenomena investigated.

The research identifies projective techniques as the most appropriate method to analyze this phenomenon, and four event managers were interviewed. Projective techniques appear innovative since they are often adopted to study people’s perspectives rather than those of managers. The results allow us to consider six different digitally contaminated PE definitions and include managerial implications.

This article is organized as follows. The first section provides a theoretical background about digital transformation and PEs. The second section describes the methodology, and the third section presents the main findings. The final section includes a critical discussion of the results and concludes with the findings.

## Background

Digital transformation is a pervasive phenomenon (Verhoef et al., 2021). It is multidisciplinary in nature, and its power lies in revolutionizing business models and consumer expectations (Lemon & Verhoef, 2016; Liu et al., 2011). Therefore, transforming processes, needs, and wants has the power to connect customers and companies in a newer and stronger relationship with consumers (Pagani & Pardo, 2017) and create innovative value propositions for customers (Berman, 2012; Li et al., 2018). In the end, companies must have digital assets, agility, networking, and big data analytics in order to achieve digital transformation (Verhoef et al., 2021), and need to orchestrate digital technologies (Lenka et al., 2017).

Digital transformation is also revolutionizing PEs (Pflaum & Golzer, 2018; Ryan et al., 2020; Dillette & Pointing, 2021). The enhancement of the digital transformation of the event industry has been boosted also because of COVID-19, considered one of the most traumatic events faced since the end of World War II (Di Maria et al., 2021, p. 297). During the pandemic, in fact, event managers immediately showed resilient behavior to face local or national closures and restrictions on gatherings. They began to innovate processes (Di Maria et al., 2021; Dillette & Pointing, 2021), significantly improving their digital skills and capabilities (Gottschalk et al., 2021). Event managers have strengthened their use of digital platforms (such as YouTube, Facebook, Instagram, Zoom, Google Meet, and others) to deliver PEs and engage audiences, allowing people to be virtually present without being physically present (Ton & Le, 2021; Vanderberg et al., 2021).

Indeed, during and after the pandemic, event managers delivered innovative events, adopting different definitions to describe them: “virtual” (e.g., Muse Enter The Simulation 2021 - see https://www.muse.mu/news/stageverse-presents-muse-enter-simulation-interactive-stadium-experience-322421), “digital” (e.g., Collision Conf 2021 - see https://collisionconf.com/blog/behind-the-numbers-2021-speakers-media-investors-startups), “hybrid” (e.g., International Marketing Trends Conference 2021 - see https://www.marketing-trends-congress.com/), and “phygital” (e.g., Cortina Ski Championship 2021). In addition, organizations are increasingly adopting the metaverse as a new environment for event delivery (e.g., Justin Bieber - The Metaverse Virtual Concert - see https://live.thewaves.network/justin-bieber/index.html).

Pearlman and Gates (2010) consider “virtual” those potential innovative spaces for corporate events and for collaborative gaming, learning, work and e-commerce. Events defined as “virtual,” according to the authors, differ from “hybrid” events because they are not a simple transposition of a “web conference” but involve the use of new platforms and advanced software. At the same time, Sox et al. (2014) state that virtual events are characterized by pervasive interactivity. Differently, a hybrid meeting is, according to the authors, the real-time overlay of features and information from physical events and virtual meetings, which allows for greater interactivity. Yung et al. (2022) suggest that while people interact only with real-world elements in physical events, in virtual environments, participants relate in real-time with synthetic computer-generated ingredients. In this sense, people participate by creating their avatars (digital replicas of the individual), far from the natural world.

Wang et al. (2019) compare in-person and digital events (such as webcasts and webinars) and conclude that while in-person events can be more distracting and time-consuming, the latter might be more effective because they are more focused on content. After investigating the differences between digital and traditional theatrical performances, Mueser and Vlachos (2018) state that several terms have been used to describe the phenomenon of live audiovisual broadcasting of performing arts, entertainment, and sports events. These include “live broadcasting,” “simulcasting,” “webcasting,” “live streaming,” “digital broadcast cinema,” “alternate (media) content cinema,” “event cinema,” “live casting,” and “relay.” The authors add that a critical difference between live streaming in public screening venues, such as movie theatres, and home viewing of live broadcasts (e.g., pay-per-view television or cable channels) or viewing on handheld devices is that public screening venues mimic the physical and social environment of traditional theatre, whether indoors or outdoors. Piccioni et al. (2021) consider “phygital events” as physical events contaminated by a mix of technologies that can be experienced immersively and interactively both physically and virtually through new technologies.

Lastly, event organizers are increasingly adopting the word “metaverse” to define their events, which are often held on gaming platforms (such as Roblox, The Sandbox, etc.). The metaverse (for which there is not yet a consensus definition in the literature - Dwivedi et al., 2022) is often considered a 3D virtual environment (Hollensen et al., 2022; Floridi, 2022; Gursoy et al., 2022), which can enable new forms of tourist consumption, experience and engagement. It involves pervasive gamification and user engagement actions (Hollensen et al., 2022).

In light of the above, essential overlaps emerge between the various categories of digitized events (such as the overlapping between virtual, digital and metaversal, as well as phygital and hybrid). This appears problematic, particularly in defining the types of impacts companies would generate on participants’ experiences and emotional responses (Yung et al., 2022). Based on the previous, it appears necessary to offer clarification for scholars and practitioners, as the terms analyzed (“digital,” “virtual,” “hybrid,” “phygital,” and “physical” and the word “metaverse”) do not have clear-cut differences. Therefore, adopting a qualitative-exploratory approach, the research aims to classify the terms, identifying distinctive elements for each.

## Methodology

### Case Study

Case study analysis is an empirical investigation that helps researchers explore contemporary phenomena (Eisenhardt, 1989; Yin, 2003). Following the Eisenhardt (1989) procedure, the researcher (a) defined the content, (b) selected the case, (c) collected and analyzed the data, and (d) wrote the results and discussions. To obtain evidence, the present study adopts a qualitative approach. In particular, case study-based techniques have been selected (Yin, 2003).

Regarding the content, the study explores a manager’s perspective on the definitions, differences, similarities, and advantages and disadvantages of in-person (physical), digital, hybrid, phygital, and virtual PEs.

The researcher selected an Italian start-up (PP) based in Milan, specializing in creating digital tools and software for event managers to achieve the objective. PP offers a highly personalized platform to manage all event operations for clients’ needs and has been awarded by the European Major Exhibition Centres Association and nominated for the best investment award of the prestigious association of English Business Angels for the best investment in the “Internet of Things” and “Smart Technologies” category.

PP was considered relevant and was selected for two other main reasons. First, PP shared the words “hybrid,” “virtual,” and “digital” via social media. Second, the company’s website reports many cases in which these words are repeated and used to indicate different kinds of PEs.

### Projective techniques

Clinical psychologists initially adopted projective techniques that have gained the scientific community’s acceptance since World War II (Bellak, 1992; Donoghue, 2010). Projective techniques are frequently assumed to understand individuals’ ways of thinking and understanding the world (Donoghue, 2010). Known as motivation research techniques (Donoghue, 2010), they help researchers understand the deeper reasons for situations (Webb, 2002). These techniques provide helpful information about the feelings, beliefs, attitudes, and motivations of people who may not be able to communicate them in a clever way or which they find challenging to express (Webb, 2002; Donoghue, 2010). They are also helpful in reducing stress and anxiety and stimulating people to say things without concern for the interviewer’s interests (Will et al., 1996), helping the interviewee overcome a host of defensive tactics (Grougiou & Pettigrew, 2011).

Consumer research frequently uses projective techniques to capture people’s hidden responses. It is advantageous if the researcher’s interest is to generate and verify hypotheses (Grougiou & Pettigrew, 2011). The disadvantages of projective techniques involve the difficulties researchers can experience in decoding and interpreting data.

As reported by Will et al. (1996), projective techniques can be categorized into four types: (a) association tasks, where the informant is requested to share with the researchers the first image, word, or thought about a specific object shown to them; (b) completion tasks, where the informant is asked to complete a sentence, story, argument, or conversation; (c) construction tasks, where respondents can say what they think about others’ actions, feelings, or attitudes; and (d) expressive tasks, where the respondent is asked to assume and act out a role or draw a concept or situation (Donoghue, 2010). These types can be used singularly or in combination to achieve the aims of the research project.

The present research explores the manager’s perspective on the definitions, differences, similarities, advantages, and disadvantages of in-person (physical), digital, hybrid, phygital, and virtual PEs. Moreover, it attempts to extend the use of projective techniques to investigate managers’ perspectives, feelings, attitudes, and motivations.

**Fig. 1:**
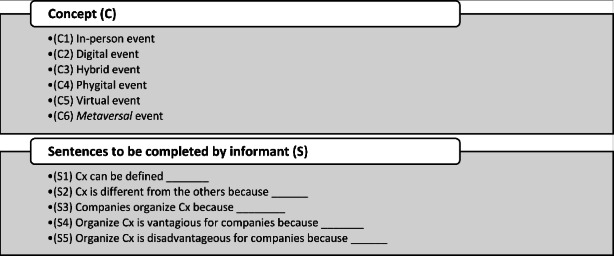
- Concepts, Sentences, and Ranking

After selecting the concept to study and given the study’s exploratory nature and the necessity of generating hypotheses for future research, the present research adopted a mix of completion and construction tasks. In the first phase, the informant was asked to complete specific sentences and rank elements depending on the concepts selected by the researcher. These concepts and sentences are reported in Fig. 1.

### Data Collection

Zoom interviews were conducted between March and August 2022; they are presented in Table 1. The interviews were recorded and transcribed, and then the researcher analyzed them. During the conversations, the researcher shared his screen, showing the informant a Microsoft PowerPoint presentation that helped share concepts by showing the sentences in Fig. 1. Other data (website information and posts) were included to triangulate the data. In addition, two senior professionals were included in the panel to obtain more comprehensive data, given the small number of employees in the company. All results are reported in the following section, including exemplary respondents’ quotes.

**Table 1:** Respondents

Code	Role	Company	Sex	Duration
I1	CEO	PP	M	35 m
I2	Strategy and Operations Director	PP	F	1h02m
I3	Event Operations Director	*Freelance*	F	21 m
I4	Institutional Relations, Digital and Event Director	*Other*	F	20 m

## Findings

In physical PEs, which must be held in a physical environment, each element of the physical location can be considered as essential as the others; catering, stages, and people are the heart of the event and contribute to generating positive vibes. Physical PEs are perceived by respondents to be the “more human” (in terms of physicality and freedom to feel and sense the physicality of the event), memorable, and essential types of events that should be considered by companies that are interested in enabling interactions and networking and giving people the freedom to do what they want in a specific place. According to respondents, physical events are preferable for engaging specific categories of audiences, delivering institutional addresses, and developing face-to-face interactions. Nevertheless, physical events are highly cost-effective, and they must face various difficulties that, sometimes, could not be unimaginable.

*I1: Physical events are those that, unique in their kind, have characteristics that none of the other types of events possesses, contact between and with people. In this event, physicality is shouted at and exalted, promoting a real relationship between individuals in a highly free form and without any constraints.*

*I3: It is, in my opinion, the most memorable event.*

*I4: I organize a physical event because I want people to meet and interact directly, creating lasting relationships which are not yet possible in the digital world.*

Digital events were considered by respondents to be a way to define virtual events. Nevertheless, according to the interviews, there were significant divergences between them. In fact, digital PEs are online occasions made by the adoption of online platforms in which people can participate by webcam, voice, or chat or by simply watching the live stream; in contrast, virtual PEs are unusual occasions where the “venue” is artificial created. In this virtual environment, people are free to manage activities, moving their avatar to explore stages, rooms, stands. Thus, digital PEs are mainly passive, while virtual PEs are highly active. Both are based on digital platforms, but while virtual events are explorable through artificial avatars, digital events do not need the creation of individuals’ alter ego. However, both allow reaching new people from around the world. Nevertheless, while virtual events are not designed to be “user-friendly” or immersive, the others offer a high immersion and allow attendees to live a not-ordinary experience. Lastly, virtual and digital events are “temporary”: when the event finishes, platforms vanish.

Digital events are highly measurable, while virtual events appear less quantifiable. However, both are less cost-effective and enable organizations to repeat events frequently. Moreover, while in digital events, people participate by their natural face or voice, in virtual PEs, they can adopt avatars and other fictional elements to represent themselves, interacting exclusively by chat.

*I1: The digital environment is more constrained; the organizer defines the parameters within which the participant can do things. This deprives the participant of any freedom, leading them to perform the intended actions upstream, barring their decision to terminate participation. […] Virtual events hide valuable data for companies, and the interactions created on the platforms can be less authentic, natural, and trustworthy.*

*I2: Digital events are only usable online. While they are more conducive to measuring results and replicability of events without too much effort in logistics, catering, etcetera, they also impose a much lower price tag on organizers and are hardly engaging for the audience. This is because, very often, following online distracts the audience, especially if they have no way to interact with the speaker or other viewers. […] It is easy to evaluate the event’s success in a digital event. […] So, these events allow you to understand how many people showed up, how they interacted, and to form an archive of what happened during the event.*

Phygital PEs were suggested to be totally different from virtual and digital events. They are physical in essence (thus organized in a physical environment) and contaminated by digital technologies. Frequently organized during the pandemic, they are supposed to be more than a simple physical event (with all its complexity) and a digital or virtual one. Digital technologies, in phygital events, are elements for creating interactions among people and between people and spaces in a seamless way. In this vein, digital technologies (such as artificial intelligence and augmented reality) are “ingredients” for enriching and enlarging reality, allowing people to move into digital or physical environments whenever they want. In addition, phygital PEs enable organizations to measure results, making them more goal oriented.

*I1: It is an event that leads to the fusion of the physical and the digital in a single space. They enable the creation of metrics even in a physical context, which is otherwise difficult to measure.*

*I4: Companies organize phygital events as much as they want that moment to be heightened, emotionally and positively, tying the brand or organization to an experience in which technology plays a key role. […] At the same time, the phygital event is disadvantageous because it requires organizers to have skills and expertise in digital management of the technologies that support the experience created in the event.*

In contrast, hybrid PEs effectively enlarges the audience without renouncing the physical dimensions of the events. In this vein, hybrid events are highly flexible and can simplify some event operations. Nevertheless, the digital component of the hybrid event is represented by the stream of the physical event (thus, the online participants watch what is happening in a specific venue). Moreover, hybrid events allow organizers to include speakers or guests from other countries since they do not need to travel and reach the physical location.

*I1: They provide for two parallel audiences, one of which watches live streaming, which broadens the audience reachable by the event.*

*I2: The hybrid event is advantageous because it allows the volume of the audience following the event to be expanded and enhanced. Therefore, it will enable greater flexibility from the participants’ side: they can choose whether to follow the event in person or online if freedom of choice is left. However, it must be considered a broadcast of the physical event.*

Finally, the metaverse is recognized as the opposite of physical PEs and as a complex environment that demands new organizers’ capabilities and competencies. Here, everything is artificial, and the environment in which people experience the event is entirely computer-generated (virtual). PEs in the metaverse are not simply virtual events but occasions delivered through specific platforms (such as Roblox and Fortnite) that could last 24 h or more, in which attendees (registered users of the platforms) are fully immersed in a different reality. When the metaversal event ends, people could continue to live metaversal experiences created by other providers, since they are “citizens” of the platform.

This type of PE is suggested to be completely innovative, totally immersive (given that the “noises” of physical experiences are fully reduced), and the future of the event industry. Moreover, *metaversal* events can rewrite event management rules; they offer new creative inputs for engaging people, interacting with them, and generating call-to-action before, during, and after the event. At the same time, some respondents emphasize the role of hardware (such as oculus or virtual reality glasses) in experiencing metaversal PEs; others remark how the hardware does not influence the fruition of the event. Additionally, in the future, the metaverse is supposed to substitute digital and virtual PEs.

*I1: Their most significant limitation derives from the technical nature of the metaverse; however, it is good to emphasize that the metaverse is a territory that companies will have to guard as a place of future conquests and explorations that will allow them to get closer to their customers and to increase the wow-effect. […] The digital event will disappear because events in the metaverse will replace it. This is because we now associate digital events with the pandemic (an alarming and dramatic period of our lives); therefore, the idea of conducting or participating in a digital event will always remind us of this period. On the other hand, the metaverse projects us into a new reality where companies can insert innovative content. So, to give an example, a webinar will no longer be in the digital realm but in the metaverse.*

*I3: The event in the metaverse breaks all the patterns we are used to knowing. It follows new, different rules that are never the same. […] For example, a rapper may hold his concert on the metaverse. In doing so, he can claim that people who want to attend the show for free have to prove that they purchased specific sneakers back in 2010. This is a completely innovative, crazy, fun selection criterion that nonetheless makes it possible for you to express.*

## Discussions, conclusions, and implications

The primary objective of the present study is to understand the differences between physical, digital, virtual, phygital, hybrid and metaversal events. In order to achieve the purpose, the work adopted a qualitative-exploratory methodology, focusing on an exemplary case of an Italian start-up. The interviews allowed for a deeper exploration of the under-investigation topics. In particular, the study adopted projective techniques to obtain meaningful data on various elements.

From the data collected, it is possible to affirm that each term (physical, digital, virtual, hybrid, phygital, metaverse) has its uniqueness. In general, however, all digitized events have a definite duration and place where they occur (whether physical or artificial) (Getz, 2008). Following previous literature (Getz, 2008; Getz & Page, 2020; Seraphin, 2021), despite the digital transformation, events continue to be perceived as social experiences that must engage participants and leave them with a memory. Regardless of the event type, the event’s ultimate objective must be to give a unique and unrepeatable moment (Getz & Page, 2020).

Through interviews, it is possible to confirm that hybrid events mean the combination of physical and digital elements, where the digital part is expressed through the replication of physical content employing digital platforms (such as Zoom, Meet, etc.) (Sox et al., 2014). At the same time, virtual events are characterized by totally artificial environments in which users move through avatars (Yung et al., 2022). However, it emerges how the concept of a hybrid event as “web conferencing” (Pearlman & Gates, 2010) is more akin to a digital event, which takes place exclusively online (Wang et al., 2019). Furthermore, research confirms how phygital events are physical events enriched by using digital technology that enables the creation of immersive and engaging experiences for users (Piccioni et al., 2021). Lastly, the study emphasizes how metaversal events are held on third-part platforms, where attendees could experience something totally different from the ordinary life, and where gamification and contestification are essential to engage people (Hollensen et al., 2022).

The originality of the research lies in comparing different categories of digitized events for the first time. In fact, the data collected allows us to state how each type of event is unique and has unique characteristics. In this sense, the terms “digital,” “virtual,” “phygital”, and “hybrid” as different. In the eyes of the respondents, they turn out to be quite distinct from each other. In addition, the term “metaverse” in events is investigated, which differs from “virtual.”

The distinction between phygital and hybrid events is primarily relevant: they are two different types of PE and have other purposes. Indeed, while phygital events are physical events contaminated by digital technologies (Piccioni et al., 2021), hybrid events merge physical and digital events to expand audiences (Sox et al., 2017).

Second, virtual and digital events are not the same. While the former requires a specific infrastructure and has peculiar elements (such as avatars), the latter is the digital transposition of a hypothetical physical event. The second, therefore, does not require the advanced skills of managers and can be organized on free digital platforms.

Thirdly, virtual and metaversal events manifest significant differences. The former involves the participation of avatars in events held in specially created artificial environments, which have a beginning and an end. On the other hand, the latter takes place on third-party platforms, in which users subscribed to those platforms participate, who are called upon to engage in highly personalized, dedicated activities aimed at the gamification of the moment (Hollensen et al., 2022).

Moreover, although in-person events are more “human,” metaversal events appear more immersive because they are less chaotic. In fact, while physical PEs are more distracting than metaversal PEs (Wang et al., 2019), the latter allows for immersive experiences through interactive components.

In addition, interviews show how the digital transformation of the event industry enables event managers to evaluate and measure event performance (Ryan et al., 2020). To assess an event, managers need to define key performance indicators and metrics in advance, selecting the most suitable type of event and a combination of digital technologies. In particular, while virtual events cover some information about user actions, digital events seem to be best suited to measure physical activities. In addition, digital events allow for basic statistics on user participation, while physical events cannot be measured in depth.

**Fig. 2:**
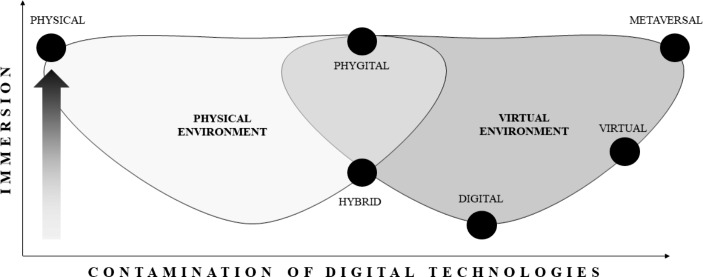
- Continuum based on contamination between PE and digital technologies or tools for participants to create event experiences

Based on the above discussion, it is possible to place all terms on a continuum, from physical to metaversal, based on the degree of contamination between PE and digital technologies or tools for creating the event experience for participants (thus excluding operations) and the perceived immersion of participants in the event experience. In this sense, physical events appear to be the least contaminated and highly immersive (although they may be *unimmersive* due to noise), while metaversal versions are the most infected, unreal, extraordinary, artificial, and immersive. Phygital events fall somewhere in between, given their physical nature and use of digital technologies to extend the real experience and high level of immersion. Hybrid events represent the sub-level of phygital PEs: they are physical occasions where digital tools are employed to replay the same content that people are experiencing in person. Digital and virtual events follow hybrid events regarding the loss of physicality, platforms that allow people to share the event freely, and a low degree of *immersiveness*. The last continuum stage is the metaversal event, in which a completely unnatural environment is mixed with physical stimulation. Figure 2 shows the continuum.

The research has both theoretical and managerial implications. On the one hand, the study clarifies the difference between various digitized programmed events, offering scholars new boundaries to define the phenomena they are investigating or aiming to explore. In particular, the work differentiates digital and virtual events from phygital and hybrid events, which are often associated or confused with each other. In addition, the research adopts projective techniques to investigate a manager’s perspective, demonstrating how these techniques can help researchers study a firm’s point of view.

On the other hand, research also allows managers to understand which event best suits the company’s goals. In particular, physical events are attractive when companies need to increase human relationships and interactions or when they want to institutionalize a specific moment or occasion. Digital events can be helpful for managers who need to limit participants’ activities, thereby increasing their focus on occasion. Virtual events are valuable in creating an immersive experience in a unique, unreal space where people are fully engaged by its interactive components. In addition, events in the metaverse can help managers increase the wow effect and rewrite the dynamics of engagement. Thus, while hybrid events, which can be hosted in physical and digital environments, enable broadening audiences, phygital events allow physical environments to be digitally transformed, enabling more precise data collection than other programmed events.

Table 2 is intended to offer managers a summary of the pros and cons of each type of event, emphasizing when it is appropriate to choose one type over another

**Table 2:** Pros and cons

	Pros	Cons
**Physical**	• Highly representative and institutional • Enables networking and face-to-face relations • Memorable	• Security and unconsidered events • Time and place defined
**Phygital**	• Measurable and goal-oriented • Engaging attendees • Amplifies the benefits of the physical event	• It needs new competencies and capabilities • Highly expensive
**Hybrid**	• Broadens event viewers and participants, even across geographic boundaries • Provides flexibility for participants and speakers	• Online participants could not feel involved in the event • The physical audience is better considered than the online audience
**Digital**	• Low costs and fully measurable • Easy to repeat in different periods • It is possible to reach an enormous number of people	• Less engaging and memorable • Disadvantageous for paid events (reduced selling prices)
**Virtual**	• Fully measurable • Immersive	• Not fully measurable • The experience could be less authentic
**Metaversal**	• Innovative and fully immersive • Highly personalized • Innovative call-to-action techniques for engaging the audience	• It needs new competencies and capabilities • Highly expensive

On a practical level, managers can benefit from the following study by considering the different aspects that have emerged. Organizing a physical event allows one to institutionalize an important moment in one’s company. In this case, it is crucial to better manage the sharing and conversation spaces dedicated to participants so that they can satisfy their need for networking. Planning a digital event could turn the audience into a passive audience. In this case, it is crucial to include engaging activities (comments, reactions, games). Making a hybrid event means expanding the pool of people reached by the event. However, it is good to think of content suitable for not excluding any audience, perhaps alternating physical and digital interventions (e.g., speakers, entertainment moments, etc.). Producing a phygital event, on the other hand, allows the physical experience of participants to be enriched. In this sense, managers should rely on engineers and professionals with knowledge of technology to build an inclusive sensory journey. Then, virtual reality, augmented reality, holograms, and emotional reading should be coordinated and orchestrated to create a unique experience for collecting valuable data. Finally, virtual events can engage selected people in a highly artificial environment. In contrast, events in the metaverse must be explicitly made, at the moment, for the subscribers to the platforms on which they take place. For this reason, a target audience analysis followed by useful surveys to understand what users are looking for in the metaverse is critical (research, in this sense, is now taking its steps).

## Limitations and further research

This study has several limitations. First, the study adopts a single-case analysis, and the number of interviews is minimal. As such, further research could expand the panel of informants. Specifically, future research could investigate event organizers and companies in a dual form.

Second, given the various elements under investigation in the study, future research could benefit from adopting a qualitative approach. At the same time, given the preliminary classification offered by the research, it might be helpful to confirm the above by administering a questionnaire to experts and managers in the field.

Third, the study uses projective techniques to collect data. In psychology and consumer studies, consumers are assumed to suffer from cultural and social biases. To avoid such biases, during the interviews, the researcher showed the respondents the six categories he was investigating; however, further research could benefit from not suggesting digitized event types (as done in the study) and trying to determine whether the practitioners recognize and know them.

Fourth, a significant limitation stems from the fact that there may be additional nuances of meaning that did not emerge. This prevents, first and foremost, conducting the interviews in Italian with native Italian speakers. Therefore, while the present study focused on an Italian start-up, further analysis could focus on English-speaking experts or realities.

Finally, the research examines only the organizer’s point of view. It would be interesting to understand the perceptions of event participants to triangulate information and capture helpful information for theory and practice.
